# 

**Published:** 1877-10

**Authors:** 


### BOOKS AND PAMPHLETS RECEIVED.

The Practitioner's Reference Book. Adapted to the use of the Physician, the Pharmacist and the Student. By Richard Dunglison, M. D. Lindsay and Blakiston. Philadelphia. Price $3.50.

Fat and Blood, and how to make them. By S. Weir Mitchell, M. D. J. B. Lippincott & Co. Philadelphia. Price $1.25.

Some General Ideas Concerning Medical Reform. By David Hunt, M. D. A. Williams & Co. Boston.

An Index of Diseases and their Treatment. By Thomas Hawkes Tanner, M. D., F. R. S. Second edition, by M. H. Broadbent, M. D. Philadelphia: Lindsay & Blakiston. Price $3.00

Personal Appearance and the Culture of Beauty, with hints as to character. By T. S. Sozinskey, M.D., Ph. D. Allen, Lane & Scott. Philadelphia.

Transactions of the College of Physicians of Philadelphia. Third series. Vol. III. Lindsay & Blakiston. Philadelphia. 1877.

Transactions of the Kentucky State Medical Society, 1877. John P. Morton & Co. Louisville.

Pathology and Treatment of Sprains. By Richard O. Cowling, A. M., M. D.

Maternal Impressions. By Thomas Waddel, M. D.

Thirty-fourth Annual Report of the Managers of the State Lunatic Asylum, Utica, N. Y., for the year 1876.

Analysis of Seven Hundred and Seventy-four Cases of Skin Disease, Treated at the Demilt Dispensary during the year 1877. With Cases and Remarks on Treatment. By L. D. Bulkley, A. M., M. D.

The Use of Obstetric Forceps in abbreviating the second stage of labor. By Edward S. Dunster, M. D.

Announcement of the Chicago College of Pharmacy, Eleventh Annual Session. October 1, 1877 to February 28, 1878.

The Strumous Element in the Etiology of Joint Disease. From an analysis of eight hundred and sixty cases. By V. P. Gibney, M. D., etc.

The Proceedings of the Medical Society of the County of Kings, Brooklyn, N. Y., August, 1877.

Case of Aneurism of the Hepatic Artery, with Multiple Abscesses of the Liver. By George Ross, A. M., M. D., and William Osler, M. D., L. R. C. P., London.

Excision of the Lower End of the Rectum in Case of Cancer. By John B. Roberts, M. D.

Contributions to the Treatment of Pulmonary Phthisis. By D. W. Gleitsmann, M. D.

Aiken as a Health Resort. By W. H. Geddings, M. D. Aiken, S. C.

Morphia in Child-birth. By W. T. Lusk, M. D. New York.

The Relations Existing between Eczema and Psoriasis. By Robert Campbell, M. D. New York.

The Toner Lectures. Lecture V. On Surgcal Complications and sequels of the continued fevers. By William W. Keen, M. D., of Philadelphia.

Nurse and Patient and Camp Cure. By S. Weir Mitchell, M. D.

Physician's Visiting List for 1878. Lindsay & Blakiston. Philadelphia.

Transactions of the Medical Association of the State of Alabama. Thirtieth session. 1877.

Retarded Dilatation of the os uteri in labor. By Albert H. Smith, M. D. Philadelphia.

Maternal Impressions. By Thomas Waddel, M. D.

Treatment of Old Dislocations of the Shoulder by subcutaneous section of the humerus and the formation of a false joint. By J. Ewing Mears, M. D.

### ANNOUNCEMENTS FOR THE MONTH.

### SOCIETY MEETINGS.

Chicago Medical Society—Mondays, Oct. 1 and 15.
Chicago Society of Physicians and Surgeons—Mondays, Oct. 8 and 22.

### CLINICS.

MONDAY.

Eye and Ear Infirmary—2 to 4 P. M., by Prof. Holmes and Dr. Hotz.
Mercy Hospital—2 to 3 P. M. Surgical, by Prof. Andrews.
Rush Medical College—1:30 P. M. Medical, by Dr. Bridge.
County Hospital—8 P. M. Necropsy, by Dr. Danforth.

TUESDAY.

County Hospital—1:30 P. M. Medical, by Prof. Bevan; 2:30 P. M. Surgical, by Dr. Bogue.
Mercy Hospital—2 P. M. Medical, by Prof. Hollister.
Eye and Ear Infirmary—2 P. M. Prof. Jones.

WEDNESDAY.

County Hospital—1:30 P. M. Gynecological, by Prof. Fitch; 2:30 P. M. Ophthalmological, by Dr. Montgomery.
Mercy Hospital—2 P. M. Eye and Ear, bv Prof. Jones.
Rush Medical College—4 P. M. Diseases of the Chest, by Prof. Ross.

THURSDAY.

Mercy Hospital—2 P. M. Medical, by Prof. Davis.
Rush Medical College—1:30 P. M. Neurological, by Prof. Lyman.
Eye and Ear Infirmary—2 to 4 P. M. Operations, by Prof. Holmes and Dr. Hotz.

FRIDAY.

Mercy Hospital—2 P. M. Medical, by Prof. Davis.
County Hospital—1:30 P. M. Medical, by Prof. Ross; 2:30 P. M., Surgical, by Prof. Gunn.

SATURDAY.

Rush Medical College—2 P. M. Surgical, by Prof. Gunn.
Chicago Medical College—2 P. M. Surgical, by Prof. Andrews and Isham; 3 P. M., Diseases of Chest, by Prof. Johnson.

Special Clinics daily, from 2 to 4 P. M., at the South Side Dispensary, and at the Central Free Dispensary.

For schedule of lectures at the colleges, apply to the college janitors.

